# Changes in the degree of lateral root trait plasticity and trade-offs of maize under long-term no tillage

**DOI:** 10.3389/fpls.2024.1345189

**Published:** 2024-02-15

**Authors:** Liming Yin, Qiushuang Lv, Peng Wang, Hongtu Xie

**Affiliations:** ^1^ Key Laboratory of Forest Ecology and Management, Institute of Applied Ecology, Chinese Academy of Sciences, Shenyang, China; ^2^ Key Laboratory of Conservation Tillage and Ecological AgricultureLiaoning Province, Shenyang, China; ^3^ College of Resources and Environment, University of Chinese Academy of Sciences, Beijing, China

**Keywords:** biomass allocation, conservation tillage, nutrient foraging, root length density, root morphological traits, root trait trade-offs

## Abstract

**Introduction:**

While no tillage (NT) can significantly influence soil structure stratification compared to conventional tillage (CT), a comprehensive understanding of the degree of root trait plasticity and trade-offs of lateral roots of crops at various growth stages along a deep soil profile in response to NT remains elusive. This knowledge gap is important for understanding soil resource acquisition strategies and yield of crops.

**Methods:**

We systematically investigated the traits of lateral roots at jointing and flowering stages in a long-term (12 years) experiment in Northeast China where maize (*Zea mays*) has been continuously planted under CT and NT with or without maize residue mulch on soil surface. We also measured soil penetration resistance and bulk density.

**Results:**

Soil penetration resistance was reduced at the jointing stage, and was increased at the flowering stage under NT especially at a depth of 10 - 40 cm. Root length density decreased under NT across the two growth stages by on average 22%. In contrast, specific root length and diameter showed greater plasticity, ranging from -14% to 20% and from -11% to 8%, respectively, relative to those under CT.

**Discussion:**

These responses could be attributed to changes in root length proportions with different diameters associated with differences in soil penetration resistance between tillage practices. The negative relationships between root traits were stronger under CT than NT, and became weaker from the jointing stage to the flowering stage. To the best of our knowledge, for the first time, our study provides empirical evidence for pivotal root trait plasticity and trade-offs across growth stages as key indicators of changes in soil structure and resources in response to NT. These insights contribute to a better understanding of soil resource acquisition strategies of crops under NT.

## 1 Introduction

Traditional soil cultivation management causes agricultural soil degradation because of excessive tillage and heavy applications of agrochemicals and has thus been considered a critical threat for maintaining crop production and food security, especially in developing countries (Geiger et al., 2010; FAO, 2013). Therefore, practical solutions for soil conservation are urgently needed for the future of human beings. In contrast to conventional tillage (CT), conservation tillage, such as minimum or no tillage (NT) with or without crop residue mulch on soil surface, has been increasingly recognized as a practical solution to maintain or even improve crop productivity, and conserve the soil ecosystem (FAO, 2013; Lehmann et al., 2020). This is because NT can reduce soil erosion, increase soil organic carbon, and decrease input costs (Blanco-Canqui and Ruis, 2018).

Compared to CT, NT provides numerous benefits for improving soil properties, which, to some extent, could be attributed to the retention of crop residue and limited soil disturbance (Giller et al., 2015; Ranaivoson et al., 2017; Sharma et al., 2018). However, long-term NT can also lead to soil structure stratification (*e.g.*, nutrient distribution and soil compaction) along the soil profile (Giller et al., 2015; Corsi, 2019) and thus can affect root development, especially of lateral roots, which is critical for soil water and nutrient uptake (Pott et al., 2020; Zhang et al., 2022). Accordingly, evaluating responses of lateral root traits to tillage practices is important to improve the ability of crop root systems to crop yield.

Previous studies have investigated the influence of tillage management on the lateral root architecture along a shallow profile (*e.g.* top 40 - 60 cm), such as root length and biomass density. For instance, Li et al. (2017a) found that soybean roots showed higher root length and biomass density in the upper layers (0-10 cm) under NT than under CT after 24 years of continuous NT practice. However, some important root traits such as biomass allocation to lateral roots and morphological traits such as root diameter and specific root length have been investigated only in a few studies despite the fact that these traits are regarded to be associated with capabilities of soil exploration and exploitation and resource absorption relative to carbon investment (Piccoli et al., 2021). More studies are needed on the effects of tillage on lateral root traits along a deep soil profile (*e.g.*, beyond 1 m).

Plasticity responses of roots to environmental changes are a pivotal strategy for plant resource foraging (Hodge, 2004; van der Bom et al., 2020). Although root trait plasticity of crop lateral roots in response to nutrient patches has been investigated (*e.g.*, Wang et al., 2023), plasticity responses to NT remain elusive. A remarkable knowledge gap on this topic prevails, especially considering that most studies have focused only on one plant growth stage, ignoring the dynamic changes in soil structure along the soil profile with plant phenology (Lynch, 2013; Dal Ferro et al., 2014). For instance, the lateral root diameter of maize can exert distinct responses to NT at different growth stages (Qin et al., 2006; Sheng et al., 2012). Thus, comprehensively investigating the degree of plasticity of lateral root traits may facilitate understanding the changes in crop nutrient foraging strategies throughout the growing season.

Furthermore, from a cost-benefit perspective, it is not necessary for crops that all root traits exhibit sensitive responses to spatio-temporal changes in soil resource associated with tillage practices (Lynch, 2015). Different root trait combinations that allow more effective exploitation of soil resource may be adopted by crops to overcome significant constraints to productivity under different tillage practices (van der Bom et al., 2020), suggesting that trade-offs and/or covariations between root traits may be dependent on tillage practice. It is increasingly recognized that the trade-offs between functional traits generally decrease with decreased severity of environmental stresses (Dwyer and Laughlin, 2017). Considering the emerging perspectives such as future roots for future soils in response to climate change and human disturbance (Lynch et al., 2022) and steep, cheap and deep to optimize water and nutrient acquisition (Lynch, 2013), there is an urgent need to identify and gain a better understanding of the trade-offs between lateral root traits involved in the efficient capture of multiple and diverging soil resources along a deep soil profile under different tillage practices.

In this study, we comprehensively measured a variety of lateral root traits, including morphology, architecture and biomass allocation at the jointing and flowering stages. We hypothesized that compared to other root traits, root diameter would have greater plasticity in response to NT likely since root diameter was particularly sensitive to changes in soil structure (*e.g.* soil compaction) (Qin et al., 2006; Sheng et al., 2012). We further hypothesized that trade-offs of root traits would become weaker from the jointing stage to the flowering stage mainly due to the improved soil structure and resources under NT (Li et al., 2017b). Overall, the aims of this study are to explore the patterns of plasticity and trade-offs of lateral root traits of maize (*Zea mays*) along a deep soil profile (up to 120 cm) at different plant growth stages in response to NT with or without plant residue mulch on soil surface.

## 2 Materials and methods

### 2.1 Study site and experimental setup

This experiment was conducted at the Conservation Tillage Research and Development Station of the Chinese Academy of Sciences, which is located at Lishu, Jilin Province of northeastern China (43°19’N, 124°14’E; Lishu station, thereafter). Lishu station was established in 2007 and maize (*Zea mays* subsp. *mays*) has been exclusively planted without crop rotations. The station experiences a temperate monsoon climate. The average annual precipitation is approximately 614 mm, mostly occurring from June to September. The mean annual air temperature is approximately 6.9°C, with the highest monthly mean temperatures occurring in July (23.7°C) and the lowest monthly mean temperatures occurring in January (-13.5°C). Soil properties are shown in **Supplementary Table S1**.

A randomized complete block design with three replicates was applied at this experimental platform. The size of each plot in each replication block was 8.7 m × 30 m, which was randomly assigned to one of the following five treatments: (1) no tillage without maize residue mulch on soil surface (NT-0); (2) no tillage with 33% (2.5 t ha^-1^, dry weight) of full amount of maize residue mulch on soil surface (NT-33); (3) no tillage with 67% (5 t ha^-1^) of full amount of maize residue mulch on soil surface (NT-67); (4) no tillage with full amount (7.5 t ha^-1^) of maize residue mulch on soil surface (NT-100); and (5) conventional tillage (CT) as the control treatment (Jiang et al., 2021). CT plots were ploughed with a moldboard plough to depths of 25-30 cm before spring sowing, and a maize residue stover was removed from the plot after harvesting in autumn. For the NT plots, the soil was undisturbed, except for maize sowing at a depth of approximately 6 cm using a no-till planter (2BMZF-4, China) with very minor soil disturbance. All plots had been subjected to CT before starting the experiment.

Maize seeds (Heyu12) was sowed on April 27, 2019, and harvest occurred on September 24 when the kernel humidity was 22%. Planting density was measured in all plots at the seedling stage (early June): 72 × 10^3^ plants ha^-1^ with a row space of 0.6 m for all the five treatments. The application rates of N, P and K were the same in all the five treatments. Mineral fertilizer (N-P_2_O_5_-K_2_O, 26%-12%-12%) was applied as basal fertilizers at seeding each year at a rate of 900 kg ha^-1^. Maize residue with a C:N ratio of 57.1 was directly and evenly distributed over the soil surface without chopping after harvest in autumn, except for the NT-0. Herbicides composed of atrazine, nicosulfuron and mesotrione were applied to all the treatment plots at the V3 stage.

### 2.2 Root sampling

For the purpose of this study, we collected root samples along the soil profile at the jointing stage (end of June, 58 days after planting) and flowering stage (middle of July, 83 days after planting) in 2019 in three treatments (with three replicates): CT, NT-100, and NT-0. Three representative plants were sampled in the middle row of each plot to avoid side effects. A soil profile was excavated at the position of mid-row perpendicularly on the right side of a given plant. Roots were then removed using a self-constructed sampler with a known rectangle surface area of 23 cm × 60 cm and to a depth that varied depending on rooting depth. We found that the rooting depth was up to 60 cm at the jointing stage and 120 cm at the flowering stage. Then samples were sliced as follows: 0-5, 5-10, 10-20, 20-40, 40-60, 60-100 and 100-120 cm. The excavated roots were washed with tap water, recovered with a stack of sieves (2 mm, 1 mm and 0.5 mm), and then frozen at -20°C until the measurement of root traits. Totally, the number of sub-samples was 135 and 189 for the jointing and flowering stage, respectively.

### 2.3 Measurement of soil physical properties

After root sampling, we collected undisturbed soil cores (diameter = 8 cm; height = 5 cm; 3 per each plot) at soil depth of 0 - 40 cm using a hand auger for soil bulk density measurement at the jointing stage. Bulk density (kg dm^-3^) was calculated by dividing the oven-dry weight (105°C for 48 h) of each soil sample by its volume. As an index of soil compaction, penetration resistance was measured *in situ* using a standard soil cone penetrometer (Soil Compaction Meter SC 900, Spectrum Technologies, Inc., Plainfield, IL, USA) with a 1.25 cm × 2.45 cm cone at the two growth stages. Data were recorded at depths of 2.5 cm, 7.5 cm, 15 cm and 30 cm. In the middle row of each plot, 10 measurements were performed randomly after soil sampling, and the mean value was calculated and used for further analysis.

### 2.4 Root characterization

We separated cleaned root samples into nodal roots and lateral roots according to position. Nodal roots were dried at 65 °C for 48 h and then weighed. However, for lateral roots that were considered highly plastic (Lynch et al., 2022), we randomly selected subsamples for scanning, while the remainder was dried. Root subsamples were arranged in trays filled with water with minimal overlap and scanned in gray scale at 500 dpi using a Microtek ScanMaker i800 Plus scanner. After scanning, these roots were also dried and weighed. From the scanned images, the average root diameter, root length and surface area were measured using WinRHIZO software (Regent Instruments Inc., Quebec City, QC, Canada). Three parts were classified according to average diameter: fine roots (< 0.2 mm), middle roots (0.2 - 0.8 mm) and thick roots (> 0.8 mm) (Li et al., 2017a). We then calculated root diameter (mm), specific root length (root length per unit dry weight, m g^-1^), specific surface area (root surface area per unit dry weight, m^2^ g^-1^), root tissue density (dry weight per unit root volume, g m^-3^) and root length proportions (root length with a given range of diameter relative to total root length, %).

Root biomass density (g cm^-3^) and root length density (cm cm^-3^) were calculated as root length, surface area and dry mass divided by soil volume, and biomass allocation to lateral roots (%) was calculated as lateral root biomass relative to the sum of lateral root biomass and nodal root biomass. For root biomass density, root length density, root diameter, specific root length, specific surface area and root tissue density we calculated trait plasticity (%) using the following equation (Fort et al., 2015; Wang et al., 2016):


$$Trait\;plasticity\;=\frac{\left(Tt\;-\;Tc\right)}{Tc}\;\times \;100$$

where T_t_ is the trait value of lateral roots in a given treatment (*i.e.*, NT-100 and NT-0), and T_c_ is the corresponding trait value in CT.

### 2.3 Statistical analysis

All data were tested for normality and homogeneity of variance. Data not meeting these assumptions were transformed using logarithmic transformation. We used a two-way ANOVA to assess the effects of tillage management and soil depth and their interaction on the traits of lateral roots, soil penetration resistance and bulk density along the soil profile at the two growth stages. Block was treated as a random effect. A two-tailed *t*-test was used to examine whether the root trait plasticity deviated from zero. Pearson correlation was used to determine relationships between root traits for each treatment. Spearman correlation analysis was performed to evaluate relationships between root length proportions with different diameter and soil penetration resistance and bulk density across treatments. The significance level was set at *P*< 0.05, and the marginal significance level was set at 0.1< *P*< 0.05. All statistical analyses were conducted with JMP (version 8.0.1; SAS Institute, Cary, NC, USA).

## 3 Results

### 3.1 Effects of tillage and depth on lateral root traits

Tillage significantly influenced root length density, specific root length, root diameter, and the length proportion of lateral roots with an average diameter between 0.2 and 0.8 mm (*i.e.* middle roots) across the two growth stages (**Table 1**). We found that the length proportion of fine roots was significantly higher, and that of middle roots was significantly lower under no tillage (NT) than under conventional tillage (CT) at the jointing stage (**Supplementary Table S2**). However, at the flowering stage, the length proportion of middle roots was significantly lower under NT than CT (**Supplementary Table S2**). In contrast, tillage did not exert a significant effect on root tissue density (**Table 1**).

**Table 1:** Two-way ANOVA results of effects of tillage and soil depth on the lateral root traits of maize at the jointing and flowering stages.

	RLD	SRL	SSA	RTD	RD	RMD	Allo	Fine	Middle	Thick
Jointing										
Tillage	0.02	0.003			< 0.0001	0.07	0.0006	0.002	0.01	
Depth	< 0.0001	0.005	0.003	0.04		< 0.0001	< 0.0001			
Flowering										
Tillage	0.007	0.0003	0.0004		0.0007				0.04	0.007
Depth	< 0.0001	< 0.0001	< 0.0001		< 0.0001	< 0.0001	< 0.0001			

RD, root diameter; RTD, root tissue density; SRL, specific root length; SSA, specific root surface area; RLD, root length density; RMD, root biomass density; Allo, biomass allocation to lateral roots; Fine, length proportion of fine roots with an average diameter of less than 0.2 mm; Middle, length proportion of middle roots with an average diameter between 0.2 and 0.8 mm; Thick, length proportion of thick roots with an average diameter of larger than 0.8 mm.

**Figure 1:**
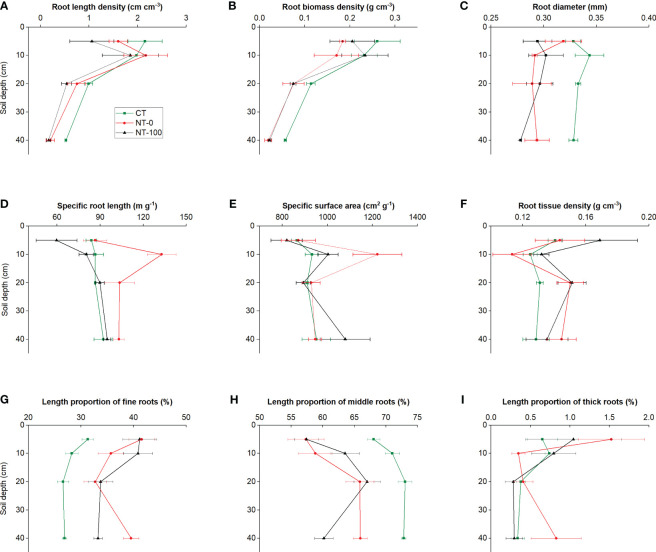
Root length density **(A)**, root biomass density **(B)**, specific root length **(C)**, specific surface area **(D)**, root diameter **(E)**, root tissue density **(F)**, and length proportions of fine **(G)**, middle **(H)** and thick **(I)** roots of lateral roots of maize along the soil profile at the jointing stage under no tillage with (NT-100) or without (NT-0) maize residue mulch on soil surface, and conventional tillage (CT).

**Figure 2:**
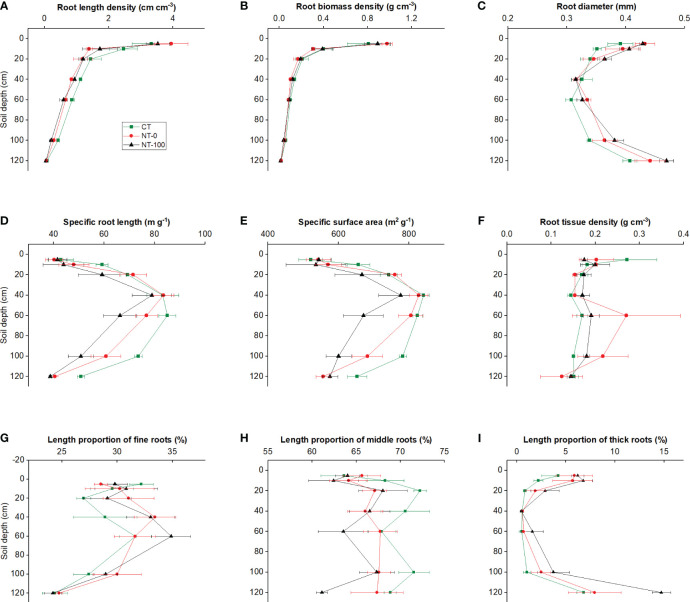
Root length density **(A)**, root biomass density **(B)**, specific root length **(C)**, specific surface area **(D)**, root diameter **(E)**, root tissue density **(F)**, and length proportions of fine **(G)**, middle **(H)** and thick **(I)** roots of lateral roots of maize along the soil profile at the flowering stage under no tillage with (NT-100) or without (NT-0) maize residue mulch on soil surface, and conventional tillage (CT).

Significantly different patterns of variation in root traits were observed with an increase in soil depth (**Figures 1**, **2**; **Table 1**). Specific root length and specific surface area increased and then decreased, whereas root diameter initially decreased and then increased at the flowering stage along the soil profile (**Figures 1**, **2**; **Table 1**). We did not find any significant interaction effects of tillage and soil depth on root traits across the two stages (**Table 1**).

### 3.2 Responses of lateral root traits to no tillage with or without maize residue mulch along soil depth

**Figure 3:**
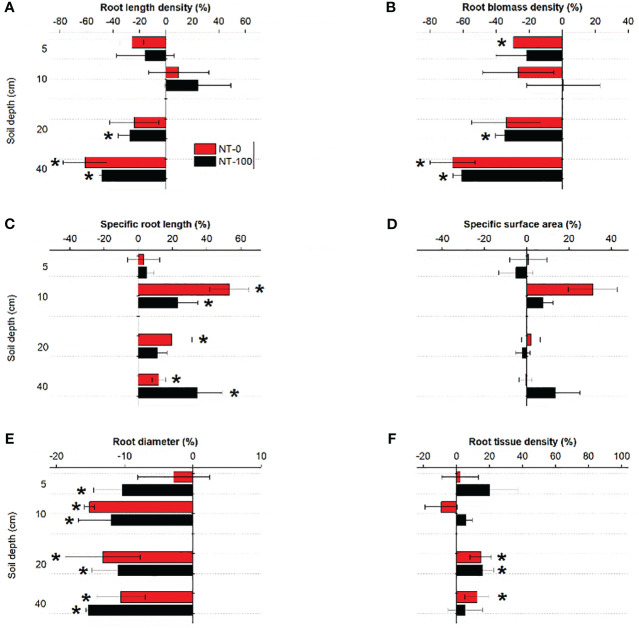
Plasticity in root length density **(A)**, root biomass density **(B)**, specific root length **(C)**, specific surface area **(D)**, root diameter **(E)** and root tissue density **(F)** of lateral roots of maize along the soil profile at the jointing stage under no tillage with (NT-100) or without (NT-0) maize residue mulch on soil surface. * denotes significant deviation from zero using two-tailed *t*-test.

**Figure 4:**
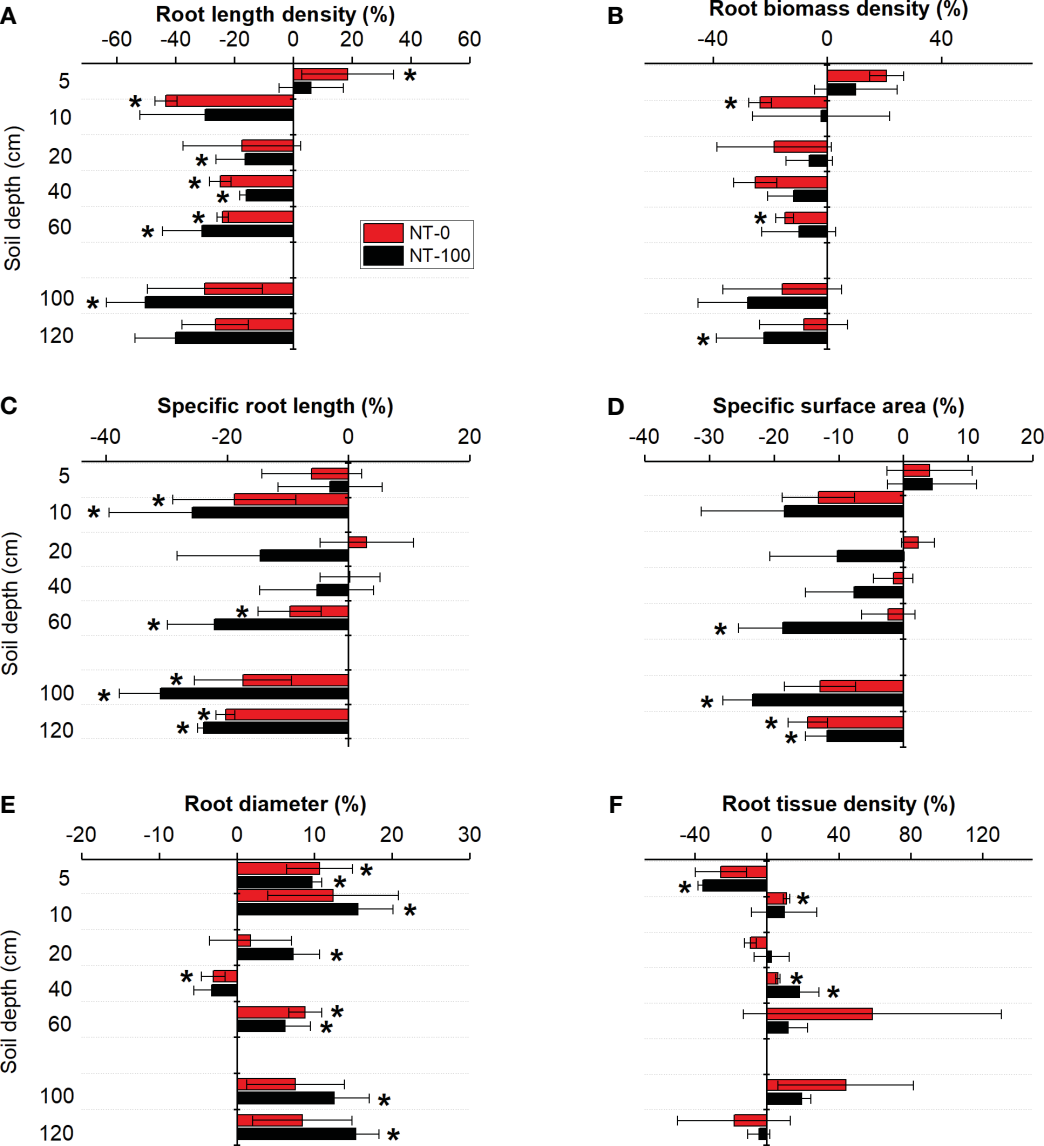
Plasticity in root length density **(A)**, root biomass density **(B)**, specific root length **(C)**, specific surface area **(D)**, root diameter **(E)** and root tissue density **(E)** of lateral roots of maize along the soil profile at the flowering stage under no tillage with (NT-100) or without (NT-0) maize residue mulch on soil surface. * denotes significant deviation from zero using two-tailed *t*-test.

We found that traits of lateral roots had varied responses to NT at the two growth stages (**Figures 3**, **4**). Plasticity of specific root length and root diameter varied largely under NT compared to those under CT along soil depth and across the two growth stages, ranging between -14% and 20% and between -11% and 8%, respectively (**Figures 3**, **4**). Root length density and tissue density exhibited remarkable plasticity only in the middle layer (10-40 cm) across the two growth stages (**Figures 3**, **4**). Compared to no tillage without maize residue mulch on soil surface (NT-0), plasticity of root traits under no tillage with maize residue mulch on soil surface (NT-100) was relatively lower across soil depth at the jointing stage but became higher at the flowering stage (**Figures 3**, **4**).

### 3.3 Effects of tillage and soil depth on soil bulk density and penetration resistance

Soil depth significantly influenced penetration resistance, *i.e.*, a significant increase with the increase in soil depth across the two growth stages (**Table 2**). We found marginally significant effects of tillage and its interaction with soil depth on soil penetration resistance at the jointing stage, especially at a depth of 10 - 40 cm (tillage * depth: *P* = 0.06, **Table 2**). At the flowering stage, compared to CT penetration resistance was significantly higher at a depth of 10 - 40 cm under NT (tillage * depth: *P* = 0.04, **Table 2**). With respect to soil bulk density, significant effects of tillage and soil depth were observed at the jointing stage (**Table 2**).

**Table 2:** Two-way ANOVA results of effects of tillage (T), soil depth (D) and their interaction (T * D) on soil penetration resistance and bulk density at the jointing and flowering stages.

Depth	Penetration resistance (kPa)						Bulk density (kg dm^-3^)		
	Jointing			Flowering			Jointing		
	CT	NT-0	NT-100	CT	NT-0	NT-100	CT	NT-0	NT-100
5 cm	335 ± 13	765 ± 278	406 ± 130	255 ± 53	112 ± 9	163 ± 22	1.60 ± 0.0	1.37 ± 0.1	1.46 ± 0.0
10 cm	1436 ± 109	1200 ± 54	1480 ± 200	805 ± 162	1274 ± 96	1178 ± 50	1.54 ± 0.0	1.50 ± 0.1	1.43 ± 0.0
20 cm	1481 ± 65	1294 ± 76	1231 ± 121	1287 ± 237	1794 ± 185	1237 ± 25	1.49 ± 0.0	1.40 ± 0.0	1.44 ± 0.0
40 cm	1425 ± 102	1193 ± 42	1109 ± 89	1758 ± 251	1595 ± 85	1080 ± 26	1.40 ± 0.0	1.37 ± 0.0	1.31 ± 0.0
Two-way ANOVA									
T	0.09			> 0.05			0.03		
D	< 0.0001			< 0.0001			0.002		
T * D	0.06			0.04			0.05		

We found significantly positive relationships between the length proportion of middle roots and penetration resistance across the two growth stages, and negative relationships between the length proportions of fine and thick roots and penetration resistance at the jointing and flowering stages, respectively (**Supplementary Table S3**). We did not observe significant relationships between length proportions with different root diameter and soil bulk density.

### 3.4 Differences in relationships between root traits among tillage treatments

**Figure 5:**
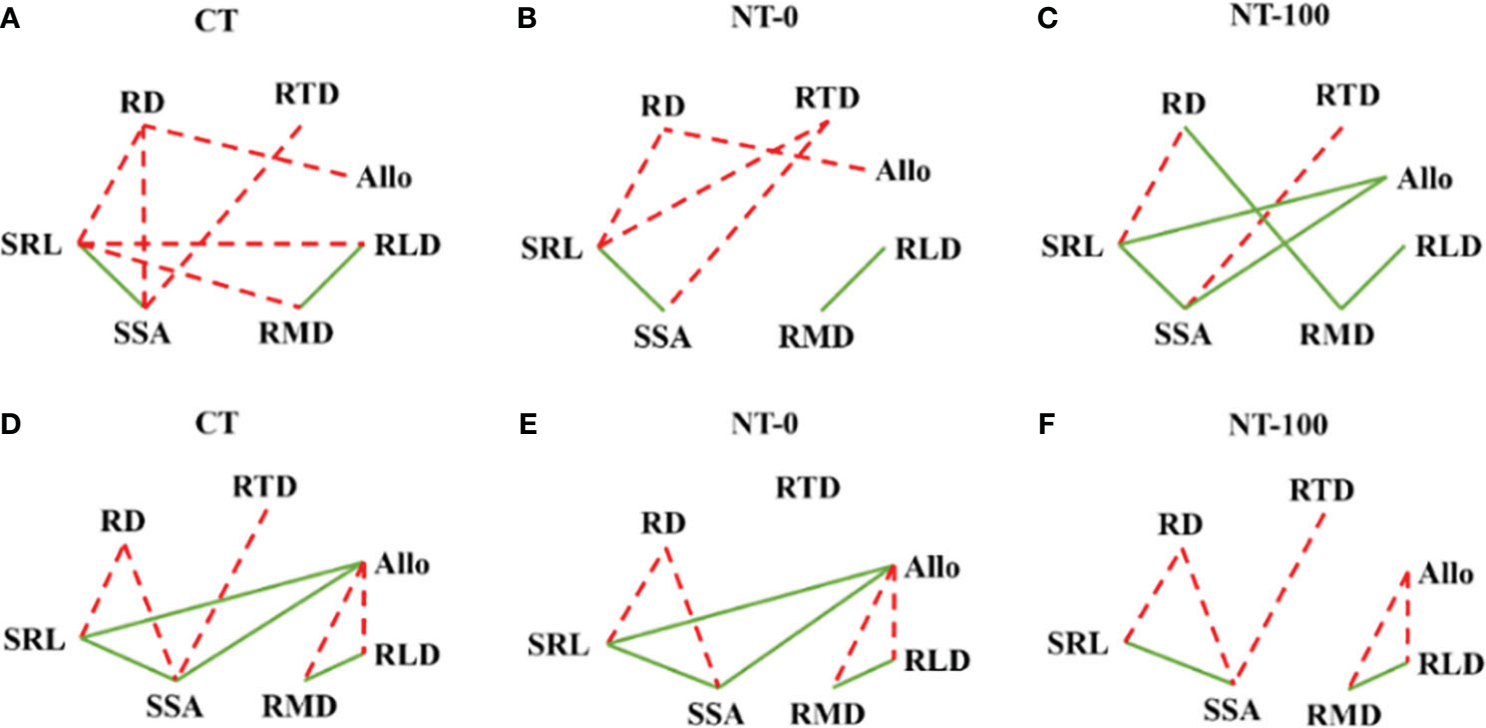
Relationship diagrams based on Pearson coefficients between root traits of lateral roots of maize across soil depths under no tillage with (NT-100) or without (NT-0) maize residue mulch on soil surface, and conventional tillage (CT) at the jointing stage (A-C) and flowering stage (D-F). Significantly (*P*< 0.05) positive (solid lines) and negative (dashed lines) relationships are shown. For abbreviations please see **Table 1**.

We found significant negative relationships between root diameter and specific root length at the two growth stages (**Figure 5**). However, some of the negative relationships between root diameter and specific surface area and between specific surface area and root tissue density disappeared under NT-0 or NT-100 (**Figure 5**). We also found significant negative relationships between root length density, biomass density and biomass allocation to lateral roots at the flowering stage (**Figure 5**). There were significant positive relationships between biomass allocation to lateral roots and specific root length and specific root surface area for NT-100 rather than NT-0 and CT at the jointing stage, suggesting that maize may strongly depend on thinner roots with higher specific root length to acquire soil resources under NT-100 (**Figure 5**).

## 4 Discussion

### 4.1 Different patterns of plasticity in lateral root traits

Our study showed large variations in six root traits at the two growth stages among tillage treatments (**Figures 3**, **4**). At the jointing stage, although root length density and biomass density were significantly influenced by tillage, their plasticity was relatively lower compared to root diameter and specific root length (**Figure 3**; **Table 1**). In contrast, root traits such as root tissue density and biomass density did not exert significant plasticity likely since these traits were conservative and mainly controlled by heredity (Bergmann et al., 2020). Thus, our findings broadly support our first hypothesis.

Interestingly, we found different changes in the direction of plasticity in root diameter and specific root length from the jointing stage to the flowering stage (**Figures 3** **4**). At the flowering stage, compared to the conventional tillage (CT) treatment specific root length significantly decreased but root diameter significantly increased under no tillage (NT) treatments with and without maize residue mulch on soil surface (**Figure 4**). A similar pattern was detected for changes in soil penetration resistance (especially at a depth of 10 - 40 cm) in response to NT from the jointing stage to the flowering stage (**Table 2**). These results suggest that responses of these root traits to NT could be attributed to no tillage effects on soil compaction and thus changes in root length proportions with different diameter. Significant relationships between the root length proportions with different diameters and penetration resistances further support this explanation (**Supplementary Table S3**). Our results are in line with the findings of previous studies where NT significantly influenced the length proportions of lateral roots with different diameters at the jointing stage (Sheng et al., 2012) and at the flowering stage (Fiorini et al., 2018; Piccoli et al., 2021).

Our findings further showed a distinct shift in the degree of plasticity in root traits across soil depth between no tillage with (NT-100) and without (NT-0) residue mulch on soil surface from the jointing stage to the flowering stage (**Figures 3**, **4**). This pattern was observed more clearly for root diameter and specific root length. However, this pattern cannot be associated with soil penetration resistance since tillage had no significant effect on it across soil depth (**Table 2**). One potential explanation could be improved soil nutrient availability (Piccoli et al., 2021), and thus a shift in nutrient acquisition strategy under NT with than that without residue mulch on soil surface (see below). Our findings suggest that a few root traits associated with root length and diameter but not necessarily all would respond sensitively to NT with or without residue mulch on soil surface. Note that almost all previous studies have focused only on traits of root length and biomass density but have ignored variations in root diameter and specific root length (*e.g.*, Fiorini et al., 2018). Taken together, this study for the first time provides empirical evidence for pivotal root traits as major detectors of changes in soil structure (and nutrient) in response to no tillage.

### 4.2 Shifts in trait trade-offs in response to NT along growth stage

Our results showed that the relationships between root diameter and specific root length, and those between specific root length and surface area were already strong under different tillage treatments across the two growth stages (**Figure 5**). In contrast, the negative relationships between root diameter and specific surface area, between root tissue density, specific root length and surface area, and between biomass allocation and root length density and biomass density varied with tillage treatment and growth stage, leading to the reduction of trait trade-offs under CT than under NT, supporting our second hypothesis (**Figure 5**). An increasingly recognized view is that enhanced trait trade-offs with environmental stress can benefit plant fitness (Damián et al., 2020). Thus, the strong reduction in trait trade-offs under NT with and without residue mulch on soil surface reflects the improvement of soil resource availability caused by the long-term use of NT compared to CT (*e.g.*, Sheng et al., 2012; Piccoli et al., 2021). However, the degree of such reduction differed between NT-0 and NT-100 along the growth stage. This result suggests that plasticity and trade-offs of root traits in response to NT depend on growth stage. The potential reasons warrant further study.

Modifications in biomass allocation is the most important response of plants to environmental conditions (Kramer-Walter et al., 2016; Wang et al., 2020). In the current study, biomass allocation to lateral roots was significantly higher, and was positively related to specific root length and specific surface area under NT-100 at the jointing stage (**Figure 5**; **Table 1**; **Supplementary Table S2**), suggesting that maize may strongly depend on thinner roots with higher specific root length to acquire soil resources. A similar pattern was reported in the previous studies (Matesanz and Milla, 2018; Wang et al., 2023) where specific root length was negatively related to the root to shoot ratio, an index of biomass allocation to roots.

In contrast, these relationships were no longer significant under NT-100 at the flowering stage (**Figure 5**). Combined with the trait plasticity of root diameter and specific root length (**Figure 4**) and no significant difference in biomass allocation to lateral roots (**Table 1**), we suggest that the soil resource acquisition strategy may shift from the “do it yourself” strategy at the jointing stage to the mycorrhizal hyphae pathway at the flowering stage, *i.e.*, the “outsourcing” strategy (Bergmann et al., 2020). NT management with residue mulch was shown to facilitate mycorrhizal fungal colonization than CT management (Hartmann and Six, 2023), although we did not measure this critical variable. Notably the relatively higher soil resource return on carbon investment for the “outsourcing” strategy than the “do it yourself” strategy could compensate for the reduced plant growth that was frequently reported at the earlier stage for NT (Fiorini et al., 2020). Thus, more attention should be paid to the role of mycorrhizal hyphae pathway in soil resource acquisition and plant growth under NT in future studies. Overall, our results suggest that clarifying the degree and patterns of trait integration under different tillage management conditions can advance our understanding of diverse crop nutrient acquisition strategies.

Using a 12-year-old experimental plantation in Northeast China, we found distinct differences in the degree of lateral root trait plasticity of maize along growth stage under NT. Specific root length and diameter showed greater plasticity than other traits. This was likely due to changes in root length proportions with different diameter associated with differences in soil compaction between tillage practices. The trade-offs between root traits were stronger under CT than under NT and became weaker from the jointing stage to the flowering stage, which reflects the improvement of soil resource availability caused by the long-term use of NT and could be associated with changes in the degree and direction of trait plasticity with growth stage. We thus highlight that sampling at various growth stages may provide more in-depth understanding of patterns of root trait plasticity and trade-offs. Furthermore, combining with measurements of mycorrhizal fungi-associated variables such as colonization, abundances, and structure may elucidate potential mechanisms of the nutrient acquisition strategy and thus crop yield under no tillage.

## Data availability statement

The original contributions presented in the study are included in the article/**Supplementary Material**. Further inquiries can be directed to the corresponding author.

## Author contributions

LY: Conceptualization, Methodology, Project administration, Writing – original draft, Writing – review & editing. QL: Data curation, Formal Analysis, Investigation, Writing – original draft. PW: Conceptualization, Funding acquisition, Supervision, Validation, Writing – review & editing. HX: Methodology, Resources, Supervision, Writing – review & editing.
