# Supplementary material for: Changes in the degree of lateral root trait plasticity and trade-offs of maize under long-term no tillage
Source: Front Plant Sci. 2024 Feb 15;15:1345189. doi: 10.3389/fpls.2024.1345189 (PMC10902851; doi:10.3389/fpls.2024.1345189)
Supplement: Supplementary file 1 [file Table_1.docx]

Supplementary Material

# Supplementary Tables

**Supplementary Table 1.** Soil properties at the Lishu conservation tillage research and development station.

| Soil properties |  |
| --- | --- |
| Sand (2–0.05 mm, %) | 28.5 |
| Silt (0.05–0.002 mm, %) | 38.6 |
| Clay (<0.002 mm, %) | 32.9 |
| Total organic C (g kg^−1^) | 11.3 |
| Total N (g kg^−1^) | 1.2 |
| C:N | 9.4 |
| Total P (g kg^−1^) | 0.38 |
| Total K (g kg^−1^) | 24.3 |
| Alkaline N (mg kg^−1^) | 90.1 |
| Available P (mg kg^−1^) | 6.9 |
| Available K (mg kg^−1^) | 143.6 |
| pH | 7.1 |

**Supplementary Table 2.** Two-way ANOVA results of the effects of tillage (T), soil depth (D), and their interaction (T * D) on length proportion (%) of lateral roots with different diameters and biomass allocations (%) to lateral roots at the jointing and flowering stages.

| Depth | Fine roots (%) | | |  | Middle roots (%) | | |  | Thick roots (%) | | |  | Biomass allocation (%) | | |
| --- | --- | --- | --- | --- | --- | --- | --- | --- | --- | --- | --- | --- | --- | --- | --- |
|  | CT | NT-0 | NT-100 |  | CT | NT-0 | NT-100 |  | CT | NT-0 | NT-100 |  | CT | NT-0 | NT-100 |
| Jointing |  |  |  |  |  |  |  |  |  |  |  |  |  |  |  |
| 5 cm | 41.5 ± 2.5 | 31.3 ± 1.1 | 41.1 ± 3.2 |  | 57.4 ± 1.9 | 68.1 ± 1.0 | 57.3 ± 2.9 |  | 1.0 ± 0.6 | 0.7 ± 0.2 | 1.5 ± 0.4 |  | 40.5 ± 7.3 | 38.8 ± 4.1 | 51.4 ± 3.4 |
| 10 cm | 35.7 ± 2.4 | 28.3 ± 1.2 | 40.8 ± 2.8 |  | 63.5 ± 2.2 | 71.0 ± 1.2 | 58.8 ± 2.7 |  | 0.8 ± 0.3 | 0.7 ± 0.1 | 0.4 ± 0.1 |  | 74.3 ± 2.7 | 65.7 ± 4.7 | 79.4 ± 3.1 |
| 20 cm | 32.7 ± 2.1 | 26.6 ± 1.0 | 33.7 ± 2.3 |  | 67.0 ± 2.1 | 73.0 ± 1.1 | 65.9 ± 2.2 |  | 0.3 ± 0.1 | 0.4 ± 0.1 | 0.4 ± 0.1 |  | 73.1 ± 2.0 | 65.0 ± 7.2 | 73.5 ± 0.8 |
| 40 cm | 39.5 ± 1.5 | 26.9 ± 0.4 | 33.2 ± 0.8 |  | 60.2 ± 1.5 | 72.8 ± 0.3 | 65.9 ± 1.1 |  | 0.3 ± 0.1 | 0.3 ± 0.1 | 0.8 ± 0.3 |  | 78.3 ± 2.4 | 57.6 ± 5.8 | 75.1 ± 1.4 |
| Flowering |  |  |  |  |  |  |  |  |  |  |  |  |  |  |  |
| 5 cm | 32.2 ± 1.1 | 28.5 ± 0.6 | 29.8 ± 1.1 |  | 63.6 ± 2.6 | 65.6 ± 2.1 | 64.0 ± 1.4 |  | 4.2 ± 1.9 | 5.9 0.6 | 6.2 ± 1.7 |  | 45.2 ± 6.8 | 42.5 ± 1.6 | 43.2 ± 4.0 |
| 10 cm | 29.5 ± 2.0 | 30.2± 3.1 | 30.8 ± 2.8 |  | 68.3 ± 2.2 | 64.1 ± 2.2 | 62.4 ± 2.9 |  | 2.2 ± 2.0 | 5.7 ± 1.0 | 6.8 ± 0.5 |  | 74.6 ± 4.3 | 66.6 ± 4.3 | 79.7 ± 5.7 |
| 20 cm | 27.0 ± 0.6 | 31.0 ± 2.3 | 29.1 ± 2.2 |  | 72.2 ± 0.7 | 67.1 ± 1.4 | 68.0 ± 2.7 |  | 0.8 ± 1.0 | 1.9 ± 1.5 | 2.9 ± 0.2 |  | 76.0 ± 4.1 | 78.6 ± 4.6 | 81.5 ± 3.6 |
| 40 cm | 28.9 ± 2.9 | 33.4 ± 1.9 | 33.0 ± 2.2 |  | 70.6 ± 2.8 | 66.0 ± 2.0 | 66.5 ± 2.3 |  | 0.6 ± 0.1 | 0.6 ± 0.1 | 0.5 ± 0.1 |  | 85.6 ± 1.9 | 84.0 ± 1.7 | 85.2 ± 2.5 |
| 60 cm | 31.7 ± 1.4 | 31.6 ± 1.9 | 34.9 ± 1.8 |  | 67.9 ± 1.6 | 67.7 ± 1.9 | 63.5 ± 2.8 |  | 0.5 ± 0.1 | 0.7 ± 1.1 | 1.6 ± 0.3 |  | 88.9 ± 1.3 | 90.6 ± 0.8 | 89.5 ± 2.7 |
| 100 cm | 27.4 ± 1.4 | 30.0 ± 2.2 | 29.0 ± 1.1 |  | 71.5 ± 1.7 | 67.6 ± 1.3 | 67.3 ± 2.0 |  | 1.0 ± 1.0 | 2.5 ± 1.6 | 3.7 ± 0.4 |  | 89.0 ± 1.2 | 93.2 ± 1.5 | 92.9 ± 1.8 |
| 120 cm | 24.3 ± 1.1 | 24.7 ± 0.3 | 24.2 ± 0.8 |  | 68.9 ± 0.7 | 67.3 ± 3.0 | 61.1 ± 0.6 |  | 6.8 ± 2.7 | 7.9 ± 1.0 | 14.7 ± 0.7 |  | 85.3 ± 5.1 | 87.9 ± 3.9 | 88.2 ± 1.4 |

Fine roots, length proportion of fine roots with an average diameter of less than 0.2 mm; Middle roots, length proportion of middle roots with an average diameter between 0.2 and 0.8 mm; Thick roots, length proportion of thick roots with an average diameter of larger than 0.8 mm.

**Supplementary Table 3.** Spearman correlation coefficients between lateral root length proportions with different diameters and soil penetration resistance.

| Stage | Length proportion of fine roots | Length proportion of middle roots | Length proportion of thick roots |
| --- | --- | --- | --- |
| Jointing | −0.42 | 0.45 |  |
| Flowering |  | 0.33 | −0.48 |

Fine roots, < 0.2 mm; Middle roots, 0.2–0.8 mm; Thick > 0.8 mm.

Significant correlations are shown (*P* < 0.05).
